# An epidemiological survey of psychiatric disorders in Iran

**DOI:** 10.1186/1745-0179-1-16

**Published:** 2005-09-26

**Authors:** Mohammad-Reza Mohammadi, Haratoon Davidian, Ahmad Ali Noorbala, Hossein Malekafzali, Hamid Reza Naghavi, Hamid Reza Pouretemad, Seyed Abbas Bagheri Yazdi, Mehdi Rahgozar, Javad Alaghebandrad, Homayoon Amini, Emran Mohammad Razzaghi, Bita Mesgarpour, Hamid Soori, Mohammad Mohammadi, Ahmad Ghanizadeh

**Affiliations:** 1 Department of Psychiatry, Psychiatry and Psychology Research Center, Tehran University of Medical Sciences, Roozbeh Hospital, South Kargar AV., 13185/1741, Tehran, Iran; 2 Director of National Research Center for Medical Sciences of Iran, No. 26, 1st Al., Kooh-e-Noor St., Motahhari Ave., Tehran, Iran; 3 Department of Biostatistics and Epidemiology, School of Public Health, Tehran University of Medical Sciences, Tehran, Iran; 4 Department of Psychology, Shahid Beheshti University, Tehran, Iran; 5 Department of Mental Health, Ministry of Health and Medical Education, Tehran, Iran; 6 Department of statistics and Computer, Social Welfare and Rehabilitation University, Tehran, Iran; 7 Iranian Center for Addiction, Tehran University of Medical Sciences, Tehran, Iran; 8 Department of Community Medicine, Medical School, Ahvaz University of Medical Sciences, Ahvaz, Iran; 9 Department of Psychiatry, Hafez Hospital, Shiraz University of Medical Sciences, Shiraz, Iran

## Abstract

**Background:**

The nation-wide epidemiological survey of psychiatric disorders in term of lifetime prevalence is not adequately known in Iran. The prevalence of lifetime psychiatric disorders was estimated among the population of aged 18 and over on gender, age group, educational level, occupational status, marital status, and residential area.

**Methods:**

The subjects were 25,180 individuals selected through a clustered random sampling method. The psychiatric disorders were diagnosed on the bases of Diagnostic and Statistical Manual of Mental Disorders-IV criteria. It is the first study in which the structured psychiatric interview administered to a representative sample of the Iranian population age 18 and over by the 250 trained clinical psychologist interviewers. The data was entered through EPI-Info software twice in an attempt to prevent any errors and SPSS-11 statistical software was also used for analyses. The odds ratios and their confidence intervals estimated by using logistic regression.

**Results and Discussion:**

The prevalence of psychiatric disorders was 10.81%. It was more common among females than males (14.34% vs. 7.34%, P < 0.001). The prevalence of anxiety and mood disorders were 8.35% and 4.29% respectively. The prevalence of psychotic disorders was 0.89%; neuro-cognitive disorders, 2.78% and dissociative disorders, 0.77%. Among mood disorders, major depressive disorder (2.98%) and among anxiety disorders, phobic disorder (2.05%) had the higher prevalence. The prevalence of psychiatric disorders among divorced and separated 22.31%; residents of urban areas 11.77%; illiterates 13.80%; householders 15.48%; unemployed 12.33% that were more than other groups.

**Conclusion:**

The mental health pattern in Iran is similar to the western countries, but it seems that the prevalence of psychiatric disorders in Iran may be lower than these countries.

It is estimated that at least about 7 millions of Iranian population suffer from one or more of the psychiatric disorders. It shows the importance of the role of the psychiatric disorders in providing preventive and management programs in Iran.

## Background

In order to make policies and strategies for control and prevention of psychiatric disorders, their prevalence in each specific community should be determined [1]. Population-based studies of psychiatric morbidity in Iran are rare. Many studies have been conducted in primary health care settings, or on specific populations and disorders [2], but these studies did not allow estimation of population prevalence.

In Iran, the previous epidemiologic studies on psychiatric disorders using Diagnostic and Statistical Manual of Mental Disorders forth version (DSM-IV) criteria have not carried out nationwide. The studies conducted in limited populations and in a few numbers of the cities that reported the prevalence of the disorders vary from 11.9% to 23.8% [3].

The only study that has been conducted is the study of the health status in Iran in 1999 [4]. 35,014 individuals at 15 years of age and over were assessed by General Health Questionnaire (GHQ-28). It showed that 21% of the subjects suffered from psychiatric symptoms. Four groups of psychiatric symptoms including depression, anxiety, somatization and social functions were studied. 879 subjects from Tehran were interviewed by psychiatric registrars based on DSM-IV criteria. The prevalence of psychiatric disorders, epilepsy, and mental retardation was estimated 21.5%. Predicted estimations of prior studies in other countries, disregard to their diversity of goals and purposes, reported up to 46% of general population [5]. These variations may be due to diversity of screening methods, diagnostic tools, sampling methods and cultural differences between populations sampled.

By estimating the nationwide prevalence of different types of psychiatric disorders, it will be useful for planning of preventive programs by health, treatment and educational authorities. This study was conducted under the title of "The national plan for epidemiologic study of psychiatric disorders in Iran."

Current study was approved by the national council for scientific research and deputy for research of Ministry of Health and was conducted by National Research Center for Medical Sciences of Iran.

The aim of the study was to estimate the lifetime prevalence rate of mental disorders and its distribution by gender, age, educational level, occupational status, marital status, and residential area in the population aged 18 years and over living in Iran.

## Methods

The lifetime prevalence of psychiatric disorders was assessed according to DSM-IV [6] by means of the Schedule for Affective Disorders and Schizophrenia (SADS) [7,8]. It is the first study ever in which a full, structured psychiatric interview has been administered to a representative sample of the Iran population aged 18 and over by 250 trained clinical psychologists. Lifetime prevalence is the proportion of the sample who reported having experienced a given disorder at some time in their lives.

### Sample

Overall, 25,180 subjects of Iranian residents, aged 18 years and over from urban and rural areas of Iran, were selected by clustered random sampling method. The country's population according to statistics provided by the health system was 63,042,188 in 1999, of which 64.5% lived in urban areas and 35.5% in rural areas. From 12,398,235 households of the country, 7,795 households were chosen. It was 1559 clusters that each one included 5 households. The choice of cluster size was based on the daily performance capacity of the data collecting group. Out of 1,559 clusters, 977 were from urban and 582 were from rural areas. The sampling framework was based on the household lists available from the department of health in the provinces. The response rate was 90%.

### Diagnostic Assessment and Instrument

The screening and diagnosis of disorders could be made based on the findings of the schedule (SADS) in one stage. The SADS questions were translated to Farsi, and then converted into English by two translators. After confirming of translation, the questionnaire was ready for the result of the test performance. To validate of the content of the questions, the test questionnaire was studied by a number of psychiatric authorities. It was applied after eliminating the defects, adjusting the main form, conducting the constructing validity, and predictive validity tests on 200 existing patients of Roozbeh hospital in Tehran, who already had a psychiatric diagnosis. On the co-rated interviews, there was a strong agreement between interviewers for diagnosis of the disorders. For test- retest interviewers, the Cohen Kappa coefficient was 0.87. The Cohen Kappa coefficients for anxiety disorders, mood disorders, and psychotic disorders were 0.79, 0.88, and 0.91 respectively. The Cohen Kappa coefficient was about 0.45 [8], in cases of substance use disorder and personality disorder. More materials about reliability, validity and specificity are in the previous articles (2, 7, 8 and 11).

### Training the use of the instrument and procedure of collecting data

The subjects were interviewed face-to-face at home by expert clinical psychologists. The interviewers were employed by the Prevention Deputy of Welfare Organization and the medical Universities in each of the Provinces. Our clinical psychologist used SADS in their interviews. In addition, they attended a three day training and role playing workshop in Tehran in order to develop proper interviewing and decision-making skills. Every clinical psychologist had to interview at least five clients and deal with the complications and questions presented in the workshop. Data gathering was precisely supervised by either a psychiatrist or psychologist and one representative of prevention deputy of each province as the supervisor of the project. The data was entered through EPI-Info software twice in an attempt to prevent any errors. The SPSS for Windows (version 11.0) used for analyzing the data. The odds ratios and their confidence interval estimated by using logistic regression.

## Results

Overall, 10.81% of subjects suffered from at least one psychiatric disorder. Major depressive disorder, phobic disorder, obsessive-compulsive disorder and epilepsy were the top four common psychiatric disorders in Iran.

The prevalence for women was about two time that of men (14.34% versus 7.34, P < 0.001). Data regarding prevalence of the psychiatric disorders in terms of age group, gender, marital status, educational level, occupation, and residential area are presented in table 1.

**Table 1:** Prevalence (%) distribution of psychiatric disorders of the overall population by the socio-demographic factors (N = 25180)

Demographic variables			Disorders			
			
		Sample(n)	Cases(n)	(%)	95% CI	P. value
**Sex**	Men	12660	929	7.34	6.9–7.8	<0.001
	Women	12520	1807	14.34	13.8–15.0	
						
**Age Groups**	18–25	7730	701	9.07	8.4–9.7	<0.001
	26–40	8196	904	11.03	10.4–11.7	
	41–55	5604	707	12.62	11.7–13.5	
	56–65	1932	196	10.14	8.8–11.5	
	>65	1659	206	12.42	10.8–14.0	
**Marital Status**	Single	7334	641	8.74	8.1–9.4	<0.001
	Married	16982	1925	11.34	10.9–11.8	
	Divorced or separated	121	27	22.31	14.9–29.7	
	Dead spouse	640	129	20.16	17.0–23.3	
						
**Residential area**	Rural	9466	886	9.36	8.8–9.9	<0.001
	Urban	15714	1850	11.77	11.3–12.3	
						
**Education**	High Education	2513	203	8.08	7.1–9.2	<0.001
	Diploma	4774	447	9.36	8.5–10.2	
	High or Guidance School	4972	459	9.23	8.4–10.0	
	Elementary	6715	784	11.68	10.9–12.4	
	Illiterate	5839	806	13.80	12.9–14.7	
						
**Occupation**	Worker	2263	172	7.60	6.5–8.8	<0.001
	Officers	2730	191	7.00	6.1–8.0	
	Student	1713	148	8.64	7.4–10.1	
	Private sector	5245	319	6.08	5.4–6.7	
	Retired	710	79	11.13	8.8–13.4	
	Housewife	9592	1485	15.48	14.8–16.2	
	Unemployed	1947	240	12.33	10.9–13.8	
	Others	909	99	10.89	8.9–12.9	

**Total**		25180	2736	10.81	10.5–11.3	

The prevalence of psychiatric disorders in the age group 18–25 was less than the other groups. Married respondents reported higher rates of lifetime psychiatric disorder than singles. Divorced, separated, and widowed respondent had higher rates of lifetime psychiatric disorder. Additionally, the rate was higher in urban areas and in the illiterate subjects (P < 0.001). In the category of occupation, housewives had the highest rate of the disorders (P < 0.001) (Table 1).

1- The prevalence of psychiatric disorders in females was more than in males except for mental retardation.

2- Married subjects had a higher prevalence rate of psychiatric disorders when compared with singles, except mental retardation.

3- The prevalence of psychiatric disorders showed a different pattern among the occupations of the subjects.

4- Educational background had no significant in prevalence of psychiatric disorder except for mood disorders, epilepsy and mental retardation.

5- The prevalence of all types of psychiatric disorders except epilepsy was higher in urban areas when compared to rural areas.

The logistic regression analysis showed on Table 2.

**Table 2:** Demographic correlation of lifetime psychiatric disorders of Iranian adults (N = 25180)

Variable	Mood Disorders		Anxiety Disorders		Psychotic Disorders		Epilepsy		Mental Retardation		Dementia	
	OR	95% CI	OR	95% CI	OR	95% CI	OR	95% CI	OR	95% CI	OR	95% CI
**Sex**												
Male	0.60	0.47–0.77	0.41	0.33–0.51	0.59	0.46–0.76	1.19	0.83–1.71	3.17	1.2–7.79	1.18	0.61–2.28
Female	1		1		1		1		1		1	
												
**Age**												
18 – 25	0.99	0.74–1.30	1.19	0.94–1.50	0.99	0.74–1.35	1.52	1.01–2.29	3.18	1.29–7.79	0.19	0.09–0.43
26 – 40	1.35	1.07–1.69	1.27	1.02–1.58	1.37	1.07–1.74	1.19	0.84–1.68	6.61	2.89–15.13	0.24	0.14–0.43
41 – 55	1.56	1.24–1.96	1.50	1.20 – 1.87	1.57	1.24–1.98	1.27	0.92–1.77	2.10	0.83–5.31	0.29	0.17–0.49
56+	1		1		1		1		1		1	
												
**Marital Status**												
Single	0.57	0.06–5.17	0.73	0.63–0.84	0.99	0.69–0.44	0.90	0.73–0.12	6.57	4.23–10.20	0.57	0.35–0.91
Widow/Widower/Separated	0.01	0.00–3.71	1.45	1.08–1.95	1.89	0.91–3.90	1.50	0.96–2.35	3.31	1.16–9.50	6.97	4.50–0.79
Married	1		1		1		1		1		1	
												
**Occupation**												
Employed	0.60	0.45–0.82	0.86	0.64–1.15	0.46	0.25–0.83	0.55	0.40–0.76	0.24	0.15–0.40	0.41	0.23–0.73
Student	0.70	0.46–1.07	0.95	0.66–1.36	0.86	0.39–1.87	0.64	0.39–1.08	0.05	0.01–0.40	0.53	0.17–1.70
Retired	0.99	0.61–1.60	1.40	0.87–2.25	0.69	0.22–2.11	0.42	0.20–0.90	0.00	0.00–18.34	0.72	0.33–1.57
Housewife	0.98	0.71–1.35	1.23	0.93–1.64	1.03	0.58–1.80	0.77	0.56–1.05	0.17	0.10–0.30	0.64	0.38–1.05
Unemployed	1		1		1		1		1		1	
												
**Education**												
University	0.59	0.45–0.76	0.88	0.65–1.18	0.88	0.65–1.20	0.50	0.33–0.78	0.02	0.01–.06	0.42	0.17–1.01
Primary and High School	0.80	0.92–0.70	1.105	0.93–1.31	0.98	0.83–1.17	0.70	0.57–0.87	0.03	0.02–0.05	0.49	0.31–0.78
Uneducated	1		1		1		1		1		1	
												
**Residential Area**												
Urban	1.26	1.10–1.45	1.48	1.29–1.70	1.43	1.00–2.06	0.99	0.81–1.21	2.05	1.34–3.16	1.71	1.16–2.52
Rural	1		1		1		1		1		1	

The odds ratios were based on logistic regression analysis.

The Prevalence rate of different types of psychiatric disorders by sex is presented in Table 3.

**Table 3:** The Prevalence of different types of psychiatric disorders by sex (N = 25180)

Type of psychiatric disorders	Males (n = 12660)		Females (n = 12520)		Total (n = 25180)	
	
	Number	(%)	Number	(%)	Number	(%)
**Mood psychiatric disorders**						
Major depressive disorder	201	1.59	549	4.38	750	2.98
Minor Depressive disorder	28	0.22	54	0.43	82	0.33
Bipolar mood disorder	23	0.18	15	0.12	38	0.96
Mood disorder with psychotic feature	3	0.02	2	0.02	5	0.02
**Total**	**255**	**2.01**	**620**	**4.95**	**875**	**4.29**
						
**Psychotic disorders**						
Schizophrenia	23	0.18	39	0.31	62	0.25
Schizoaffective	9	0.07	16	0.13	25	0.10
Short term psychotic disorder	6	0.05	5	0.04	11	0.04
Schizopherniform	4	0.03	2	0.02	6	0.02
Other psychotic disorders	43	0.34	78	0.62	121	0.48
**Total**	**85**	**0.67**	**140**	**1.12**	**225**	**0.89**
						
**Anxiety disorders**						
Panic disorder	100	0.79	274	2.19	374	1.49
Generalized anxiety disorder	93	0.73	243	1.94	336	1.33
Obsessive compulsive disorder	91	0.7	353	2.8	444	1.8
Agoraphobia	34	0.27	141	1.13	175	0.70
Phobic disorders	113	0.89	403	3.22	516	2.05
Post traumatic stress disorder	98	0.77	150	1.20	248	0.98
**Total**	**529**	**4.15**	**1564**	**12.48**	**2093**	**8.35**
						
**The neuro-cognitive disorders**						
Epilepsy	199	1.57	255	2.04	454	1.80
Mental retardation (Severe)	56	0.44	51	0.41	107	0.42
Dementia	65	0.51	75	0.60	140	0.56
**Total**	**320**	**2.52**	**381**	**3.05**	**701**	**2.78**
						
**Dissociative and Somatization disorders**						
Somatization Disorder	16	0.13	30	0.24	46	0.18
Dissociative Fugue	3	0.02	5	0.04	8	0.03
Dissociative Amnesia	65	0.51	61	0.49	126	0.50
Depersonalization Disorder	7	0.06	7	0.06	14	0.06
**Total**	**91**	**0.72**	**103**	**0.83**	**194**	**0.77**

**Total**	**1280**	**10.07**	**2808**	**22.43**	**4088**	**17.08**

Each subjects may suffered from more than one of the above psychiatric disorders.

## Discussion

Most of the subjects (89.2%) lack any psychiatric disorder. The prevalence of subjects with no lifetime DSM-III-R diagnosis in a study in Oslo was 47.7% [9]. Also, the rate in the National Co-morbidity Study in the USA was 52% [10]. Although the two study samples were taken from the general population, they used the Composite International Diagnostic Interview [11] and DSM-III-R criteria. This difference may be based on the instrument and method used in their studies. For example, the Norway study focused on the population of Oslo, a large city with different social problems when compared to small cities or villages. This generalization cannot be applied to the whole country. It is possible that some depressive and anxiety disorders are more common in larger cities with a higher rate of disorder. The National Co-morbidity Survey in the United States [9] was a large cross-sectional population study with a design that was not comparable to our study. Also, the US study was limited to respondents aged 15–54. Plus, they used a paper and pencil version. These differences in the psychiatric interview may be responsible for the differences in the findings.

A cross national study showed that one third of the subjects experienced at least one disorder at some time in their life in Brazil (36.3%), Canada (37.5%), Germany (38.4%), Netherlands (40.9%), and the USA (48.6%). Lifetime prevalence estimates were considerably lower in Mexico (20.2%) and Turkey (12.2%) [12]. The current study shows that psychiatric disorders are not infrequent in Iran. As many as 10.81% of the subjects reported having experienced one or more psychiatric disorder at some time in their lives. However, it is lower than the rate reported in the study that used GHQ-28 [4]. The difference may be due to the method and tool used for screening and diagnosis of the disorders. The SADS included 16 groups of psychiatric disorders whereas the GHQ-28 questionnaire only studies the symptoms of anxiety, depression, somatisization and social dysfunctions.

The present study shows that the rate of psychiatric disorders in women is higher than men (14.34% versus 7.34%) which are consistent to the results of some studies conducted in Iran and other countries [4,9,13,14]. However, a study in Netherlands, reported that more than four out of ten respondents (41.2%) reported a lifetime prevalence of at least one DSM-III-R disorder. The most common psychiatric disorders were major depression, alcohol abuse and phobias with no significant difference between men and women (42.5% versus 39.9%). More than 15% of the respondents had major depression in their history. Though they did not find gender differences in the overall prevalence of mental disorders, differences did emerge when the disorders were examined separately. Obsessive-compulsive disorder was the only exception among the anxiety disorders [15] that was more prevalent among men. In the current study, all types of the psychiatric disorders were more common in females than males except BMD, acute brief psychotic disorder, somatoform disorder, and amnestic disorder.

In a study in Brazil based on ICD-10, it showed that nearly 46% of the sample had at least one lifetime mental disorder, i.e., almost one in two respondents reported a given disorder at some time in their lives [5]. They reported that women were more likely than men to have mood disorders (with the exception of bipolar disorder, for which there were no gender differences), and anxiety disorders (except for obsessive-compulsive disorder, social phobia, and generalized anxiety disorder). There were no gender differences in the rates of somatoform disorders.

The results of the some previous studies showed a different pattern, with the highest prevalence typically occurring among the youngest age groups [[12,16], and [17]]. The current study shows that the prevalence of psychiatric disorders in the ages of 41 and over is more than the age group 18–40. A study in Norway showed that the most common age group was 30–39 [9]. The rate in the USA was more in the age group 25–34 [10].

The highest estimated prevalence was found among respondents at the lowest level of educational attainment. This is in accordance with results of six of the seven surveys of the cross national study (Canada, USA, Brazil, Mexico, Germany, Netherlands, and Turkey). Germany was the exception (with an insignificant relationship) [12]. Also, it was consistence with the study in Norway [9].

Some studies have found an occupational gradient in the prevalence of common psychiatric disorders [18]; others have failed to find such association [19]. Our result is similar to the later study. A study in Brazil using ICD-I0 classification showed that except for anxiety disorders, unemployed respondents were more likely to have any lifetime disorder. Students, homemakers, and retired persons were all less likely to have any psychiatric morbidity [5] when compared with the employed.

The rates of most types of psychiatric disorders in urban areas are higher, when compared to rural areas. These findings are reinforced by the previous study [3]. Recently, a study showed that the difference was not statistically meaningful [20]. It is possible that other factors that are related to location of residence are more important, such as: poverty, unemployment, lower socioeconomic status, and sex.

The differences in the methods of selecting the samples, operational definition of variables, data gathering methods, and tools are considered as important factors in inconsistencies with the results. In particular, the validity and reliability of the tool should be considered. The validity and reliability of SADS was well reflected in the previous studies [21,22]. However, the validity and reliability of western societies does not confirm its validity in countries with deep cultural difference. The effects of cultural factors on estimating the prevalence of psychiatric disorders through diagnostic interviews has been shown in previous studies [23]. The present study shows that the prevalence rate of bipolar mood disorder (BMD) was 0.96%, the results of the other studies showed a different pattern. Findings reported by the recent multi-center European study prevalence rate of BMD reveal even lower frequencies under 1%. Data from surveys of large samples showed the lifetime prevalence rates of BMD around 1.5%. A main question is whether the low prevalence rates of BMD are not an artifact of the over diagnosis of depression [24].

## Limitations

The community epidemiological surveys of psychiatric disorders have been carried out in different parts of the world in the absence of a common format for diagnostic interviews. Therefore, cross-national syntheses or comparisons of the results of these surveys could not be made.

Substance use is one of the major concerns of mental health in Iran, and was not addressed in the current study. In cases of substance use disorder, the Cohen Kappa coefficient was low. It may be due to the expensive penalty for substance use in Iran e.g. loss of job. Another limitation includes difficulty in recalling the past, which may cause under reporting. The study relied only on self-reporting to make diagnosis. As well, it is possible that some respondents in community surveys did not disclose information about psychiatric disorders to interviewers. Also, we know that a substantial portion of people with relatively uncommon disorders like schizophrenia are already long-term residents of mental hospitals, and thus may have not been included in the study. Unfortunately, there has not been any survey of people with schizophrenia in Iranian hospitals. This unwillingness of disclosure may not take a form of high rates of refusal to participate in epidemiological surveys, but rather of high rates of agreement accompanied by intentionally low reported rates of disorders. A noticeable population of Iranian people has immigrated to the other countries during last 3 decades and we don't know how this could be influencing the results. Prevalence rate may be affected by recall bias for episodes of nervous problems years before. Less severe episodes of symptoms earlier in life may be forgotten. Forgetting episodes may not be just a matter of forgetting, but also a result of processing the significance and meaning of symptoms with time. It is questionable whether symptoms that today are considered medical would now be understood and reported as nervous symptoms. These types of biases could be effective in interpretation of the results. It is possible that the diagnostic assessment and the instrument was not the most accurate method for this study. This instrument (SADS) was chosen over others (SCAN, DISC, CIDI, SCID and etc.) because of its wide use of it in Iran (for example; Mohammadi et al. [2,8]). The experts are more familiar with SADS which is used by our clinical psychologist in their daily clinical practices while K-SADS is used in Iran for children and adolescents. Without any bias the lifetime prevalence should increase clearly by increasing age. In contrary, the prevalence seems to be lowest in those over 56 years of age. Finally, this article does not address the one year or six month prevalence of the disorders that might have been more useful for making policies and strategies for the prevention of the psychiatric disorders.

## Conclusion

The pattern of prevalence of mental health in Iran is similar to the western countries, but it seems that the prevalence of psychiatric disorders in Iran may be lower than these countries.

Women along with retired and unemployed men had more types of psychiatric disorders, mandating a necessity to plan programs in prevention and treatment of these disorders.

On the bases of the results of the current study, it is estimated that at least about 7 millions of Iranian population suffer from one or more of the psychiatric disorders and they need mental health services. It shows the importance of incorporating psychiatric disorder in planning preventive and management programs in Iran.

## Competing interests

The author(s) declare that they have no competing interests.

## Authors' contributions

Mohammad-Reza Mohammadi (principal investigator, conception, designer, country coordinator and final approval, psychiatrist)

Haratoun Davidian (designer, psychiatrist)

Ahmad Ali Noorbala (conception and design, psychiatrist)

Hossein Malek Afzali (statistical consultant, biostatistician)

Hamid Reza Naghavi (designer, province coordinator and investigator, psychiatrist)

Javad Alaghebandrad (designer, coordinator and investigator, psychiatrist)

Seyed abbas Bagheri Yazdi (interpretation and revising, clinical psychologist)

Mehdi Rahgozar (statistical analysis, programmer, interpretation and revising, biostatistician)

Hamid Reza Pouretemad (province coordinator and investigator, clinical Psychologist)

Homayoon Amini (province coordinator and investigator, psychiatrist)

Emran Mohammad Razzaghi (investigator, psychiatrist)

Bita Mesgarpour (coordinating and revising, pharmacist)

Hamid Soori (statistical consultant and revising, epidemiologist)

Ahmad Ghanizadeh (interpretation and revising, psychiatrist)

All authors read and approved the final manuscript.
